# Accurate Localization in Acoustic Underwater Localization Systems †

**DOI:** 10.3390/s21030762

**Published:** 2021-01-23

**Authors:** Gianni Cario, Alessandro Casavola, Gianfranco Gagliardi, Marco Lupia, Umberto Severino

**Affiliations:** 1 Dipartimento di Ingegneria Elettronica, Informatica e Sistemistica (DIMES), Universitá della Calabria, 87036 Rende (CS), Italy; g.cario@dimes.unical.it (G.C.); g.gagliardi@dimes.unical.it (G.G.); m.lupia@dimes.unical.it (M.L.); 2 Dipartimento di Ingegneria Meccanica, Energetica e Gestionale (DIMEG), Universitá della Calabria, 87036 Rende (CS), Italy; umberto.severino@unical.it

**Keywords:** extended kalman filter, underwater localization, acoustic ranging, positioning systems, error analysis, transponders

## Abstract

In underwater localization systems several sources of error may impact in different ways the accuracy of the final position estimates. Through simulations and statistical analysis it is possible to identify and characterize such sources of error and their relative importance. This is especially of use when an accurate localization system has to be designed within required accuracy prescriptions. This approach allows one to also investigate how much these sources of error influence the final position estimates achieved by an Extended Kalman Filter (EKF). This paper presents the results of experiments designed in a virtual environment used to simulate real acoustic underwater localization systems. The paper intends to analyze the main parameters that significantly influence the position estimates achieved by a Short Baseline (SBL) system. Specifically, the results of this analysis are presented for a proprietary localization system constituted by a surface platform equipped with four acoustic transducers used for the localization of an underwater target. The simulator here presented has the purpose of simulating the hardware system and modifying some of its design parameters, such as the base-line length and the errors on the GPS and Inertial Measurement Unit (IMU) units, in order to understand which parameters have to modify for improving the accuracy of the entire positioning system. It is shown that statistical analysis techniques can be of help in determining the best values of these parameters that permit to improve the performance of a real hardware system.

## 1. Introduction

In the last few decades, interest in research on underwater systems has been large. In particular, many advancements have been achieved on the electronic systems that support the localization of underwater vehicles and divers. The research on underwater vehicles has focused mainly on AUVs (Autonomous Underwater Vehicles), that are now routinely used for many tasks such as SAR (Search And Rescue), environmental and biological monitoring, seafloor mapping, oil and gas exploration, and underwater infrastructure monitoring. Research on diver electronic support systems has focused on safety systems, like dive computers, and Underwater Augmented Reality (UWAR) systems that provide visual aids to increase the capability ofcommercial divers to detect, perceive, and understand elements in underwater environments [1]. UWAR systems are used also for recreative applications such as underwater exploration of archaeological underwater parks [2,3]. In order to improve those tasks, accurate localization and navigation become extremely important to guarantee the correctness of the gathered data for these applications [4].

On the surface, most embedded localization systems use GNSS (Global Navigation Satellite System) like (GPS, GLONASS, Galileo, Beidu, etc.). However, these systems use radio links that propagate only at a very short distance underwater. In this environment, the most used localization techniques are based on acoustic signals [5]. The target that needs to be localized uses the distances among three or more transponders at known positions. The distances, or ranges, are calculated by exchanging acoustic signals between each transponder and the target. Two approaches are used for this purpose, both are based on the computation of the acoustic signal Time of Flight (ToF): The One-Way Time Travel (OWTT) or the Two-Way Time Travel (TWTT). The first one needs the transponders and target clocks to be synchronized, the latter does not require this assumption. The possible configurations [6] of a localization system depends on the mutual position of each transponder: Long Baseline (LBL), Short Baseline (SBL), Ultra Short Baseline (USBL) acoustic positioning systems are the most common. Each configuration has different characteristics in terms of the boundary conditions of the scenario and performance [7]. In the LBL case, a set of acoustic transponders is pre-deployed on the seafloor around the boundaries of the area of interest and the distance among transponders is typically tens or hundreds of meters. In SBL systems, a set of three or four transponders are installed on a fixed rigid structure under a mother ship or on the seafloor. In this case, the distance between the transponders is at most a few meters. The receiver gets its absolute position via bidirectional acoustic communication with the transponders as in LBL systems. In USBL systems, the transducers are built into a single transceiver assembly or an array of transducer elements casted in a single transceiver. The distances are measured by differences arising in the “time-phase” of the signal in each element of the transceiver array with respect to a reference. The “time-phase differences” among transducer elements are also used to compute the bearing of the transmitted signal by the receiver [8].

Ideally, it is preferable that the underwater acoustic localization systems performs in the same way as the surface localization systems based on GPS. The underwater acoustic localization is challenging due to many limitations of digital acoustic communications like small bandwidth, low data rate, variable sound speed, and data loss. Applications of acoustic localization can be found in several fields of interest: Geophysical field surveys, inspection of offshore plants, environment monitoring, localization of points of interest, and acoustic navigation. The accuracy of the localization systems is crucial and localization errors must be small in proportion to the complexity of the scenario in which the assets are deployed.

The aim of this paper is to present a simulation approach through which, for a given set of error sources, it is possible to know how much they affect the total localization error. The estimation of the total localization error and the definition of the parameters of interest (which form the total position error) are crucial steps to analytically and experimentally study how the total error is influenced by the single sources. Such a kind of analysis permits one to understand the sources that have the main influence and to take actions to improve them by providing an indication of the current maximum achievable accuracy. More practically, a better knowledge of the system and its characterization in a virtual design environment allows the designers of low-cost localization systems to reduce the development times by focusing their attention and efforts onto the optimization of the main parameters of influence.

As an example of application, we consider the following scenario: A target diver is following an underwater path or is moving through two defined points and they need to be accurately localized during their motion. To this end, we use an SBL localization system with two main components: The SBL base and the SBL receiver. The SBL base is a surface platform, equipped with four acoustic beacons, a GPS receiver, and an Inertial Measurement Unit (IMU) device. The SBL receiver is an underwater device used by the target diver to compute their position with respect to the SBL base. The SBL receiver gets the acoustic signals from the SBL base, computes the distances from each beacon, and implements the localization algorithms fusing all available data. The communication between the SBL beacons and the target receiver is accomplished by an acoustic modem (in particular, the SeaModem developed by Applicon S.r.l (Applicon S.r.l. is an engineering company, spin-off of the University of Calabria, dealing with the hardware/software design and development of microprocessor-based embedded systems for all needs of communication, networking, localization, navigation, monitoring and remote control of vehicles, sensors, plants, and divers in underwater environments) has been here used). All of these devices are source of errors that contribute to the total localization error in different percentages.

Many details regarding sensors and localization techniques have not been provided as the hardware system (SBL base + SBL receiver) is under patent application. The paper intends to analyze the main parameters that significantly influence the position error estimated by the system with respect to the real one. The simulator therefore has the purpose of simulating the hardware system and modifying some of its parameters, such as the base-line length and the error on the GPS and IMU units, in order to understand how to act to reduce the accuracy error of the entire positioning system.

Some preliminary results of this work have been published in [9]. In this respect, this paper represents an extended version containing enlarged domains of applicability, additional technical details on the practicability of the proposed simulation environment, and a comprehensive set of simulations, allowing the assessment of the approach, carried out with data acquired from real diving sessions.

The paper is organized as follows: Section 2 describes the real application scenario and its related sources of error. The Extended Kalman Filter (EKF) is discussed in Section 3. Section 4 concerns with details related to the construction of the simulator and its interaction with the EKF. The outputs of the simulator are matched with the outputs of the real system in Section 5. In Section 6 the simulation and statistical analysis and concluding remarks are presented.

## 2. Real Application Scenario

**Figure 1:**
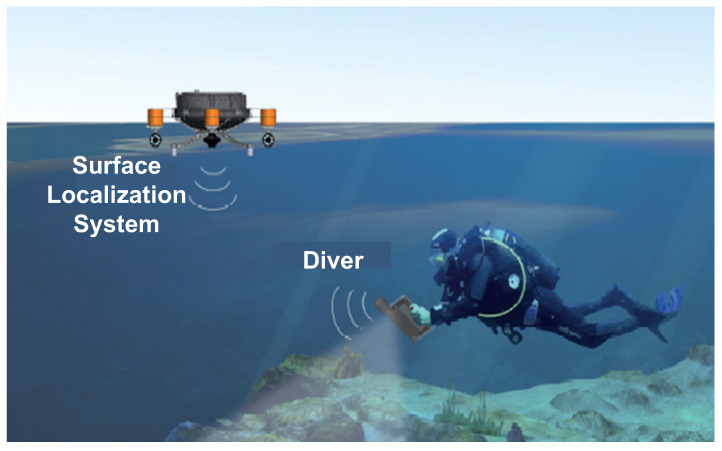
Real application scenario.

Applications of underwater acoustic localization system can be found in several fields. In this paper we account an application focused on divers localization in underwater archeological sites. The divers are equipped with an underwater tablet embedded with an acoustic transducer and a depth sensor (see Figure 1).

Both the divers and the surface localization system exploit the SeaModem [10]. A signal is emitted sequentially from each beacon of the localization system and a round-robin scheduling logic is used to process the incoming data. The round-robin scheduling periodically runs a job for a time slice and then switches to the next job in the run queue. It repeatedly does so until the jobs are finished and then starts again from the first job of the queue [11]. The depth sensor measurement is acquired at each time step. The SeaModem receiver of the underwater tablet acquires the acoustic signals containing the identification number of the beacon, the North-East-Down (NED) coordinates of the beacons and/or the epoch at which each signal is transmitted. Receiver and transmitter are clock-synchronized and it is possible to compute the four ranges at different time instants with a One-Way Time Travel technique.

If the times at which the signal is transmitted and received and if the speed of sound in the medium are known, it is possible to compute the distance between the transmitter and the receiver. The speed of sound in seawater depends on characteristic parameters such as temperature, salinity, and depth [12]. There are several physical models to estimate the speed of sound but all of these introduce an approximation error in the estimates [13]. The sound speed used in the simulator was set to 1500 m/s. As described in Section 2.3, the acoustic hardware under development can measure the sound speed through a built-in procedure present in the SeaModem by knowing the exact distance between two modems. Typically, the sound speed is measured by putting the transmitter and a receiver in the arbor 10 m apart. The interval between two successive beacon acquisitions depends on the guard-interval that are used to ensure that distinct transmissions do not interfere with one another, or otherwise cause overlapping transmissions to avoid mutual interferences in the transmissions. The guard-interval influences the sampling time of the measurements and, due to slightly non-congruent information among successive range acquisitions, introduces a proportional error in the position estimates. Furthermore, packets loss and outliers have to be dealt with because of reflection phenomena due to the environment.

**Figure 2:**
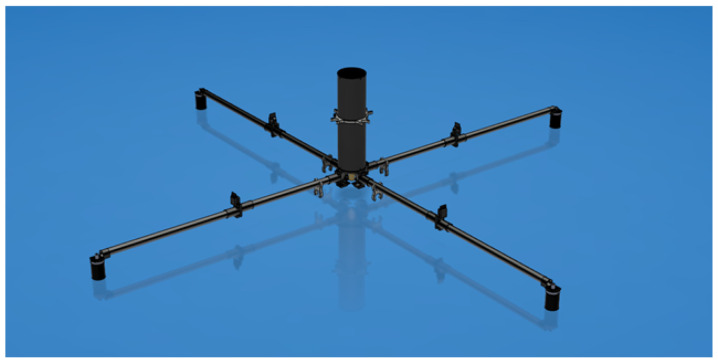
Standalone Short Baseline (SBL) system with four beacon transducers. Virtual prototype.

For each configuration, it is possible to achieve different results in localization. These different results also depend on the length of the baseline and the known position of the system. In our application, the target establishes its own position with respect to the frame of the localization system. Both the LBL and the SBL systems are geo-referenced. A SBL system could be attached to a surface vehicle and the position of the centroid of the cross constituted by the four beacons (Figure 2) is known via a GPS measure. An Inertial Measurement Unit (IMU) measures the orientation of the SBL. If the beacon positions are not fixed, e.g., subject to the waves motion, the IMU provides information for the correction of the positions of the beacons. The GPS and the IMU introduce an error due to the accuracy of the instrumentation [14]. For civil applications (Standard Positioning Service, SPS) the accuracy of the GPS was limited. GPS accuracy could be increased through further techniques (dual-frequency, augmentation systems). A focus on the main sources of error are hereafter reported.

### 2.1. GPS Error Sources

**Figure 3:**
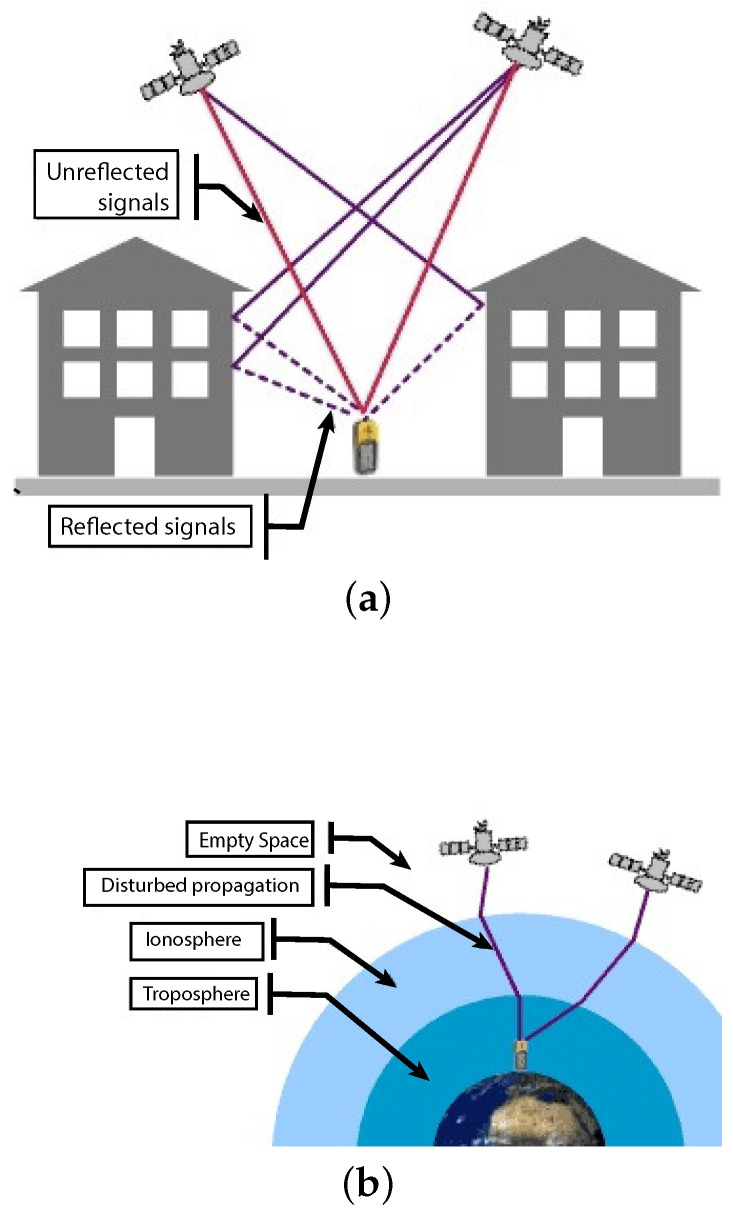
GPS error sources: Multipaths (**a**) and Atmospheric Signal Refraction (**b**).

The GPS error sources (see Figure 3b) can be user dependent and user independent. Most errors depend on factors such as [15]:

- Atmospheric conditions;
- Satellites geometry;
- Satellites signal blockage (buildings, bridges, trees, rocks, etc.);
- Receiver design features and quality;
- Indoor or underground applications;
- Signals reflected off buildings, walls, or water.

The position calculated by a GPS receiver requires the current time, the position of the satellites, and the measured delay of the received signals. In the calculation of the signal delay, the error due to the transmission is about 3 m because of the speed of propagation of the signal [16,17,18], but it can be reduced by a factor of 10 by using the higher-chip-rate P(Y) signal [17]. As an example, the GPS receiver accuracy used in consumer products (e.g., smart-phones) has a value of 4.9 m [19]. The GPS receiver used in our application has an accuracy between 0.1 and 3.5 m, that is the accuracy expected for the SBL system under development. An error on the GPS positioning affects the computation of the position of the beacons. The NED coordinates of the beacons are received from the target and are used to estimate its position via the EKF filter. The effects of a non-precise knowledge of the beacons positions impact on the resulting accuracy of the localization system [20]. In our system, the GPS is also used to synchronize the clocks of the transmitter and the receivers. In fact, GPS time transfer is a common method for synchronizing clocks and networks to Coordinated Universal Time (UTC). This synchronization uses GPS signals and its accuracy is between 0 to 40 nanosenconds [19]. Note that the SBL receiver used by a diver uses the GPS signal only at the power-on to synchronize its clock. When the diver is underwater, the GPS receiver is unused and only the internal clock maintains synchronization. The drift in clock synchronization affects the distance calculated via the One-Way Time Travel protocol.

### 2.2. IMU Error Sources

**Figure 4:**
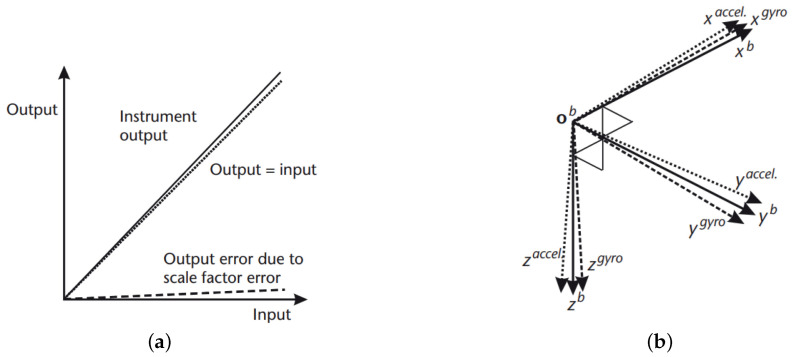
IMU error sources: Scale factor (**a**) and misalignment of accelerometer (dotted lines) and gyro (dashed lines) axes (**b**).

All types of accelerometers and gyros exhibit biases (Table 1), scale factors (Figure 4a), misalignment (Figure 4b), and random noise to a certain extent [21]. Higher-order errors and angular rate-specific force cross-sensitivity may also occur, depending on the sensor type [6]: Spinning mass, vibratory, and some fiber optic gyros exhibit a g-dependent bias due to mass unbalance. Some ring laser and micro-electromechanical system (MEMS) gyros at a high angular rate exhibit a non-linear scale factor variation. Each IMU error source is composed by four components that affect the total error: A fixed contribution, a temperature-dependent variation, a run-to-run variation, and an in-run variation. The fixed contribution is a constant error present each time the sensor is used. The temperature-dependent component is an error due to different mechanical response at different temperatures. The run-to-run variation results in a contribution to the error source that is different each time the sensor is used but remains constant within any run. Finally, the in-run variation contribution to the error source slowly changes during the course of a run.

The run-to-run variation can be calibrated by alignment and integration procedures each time the IMU is used. The in-run variation is difficult to correct without another navigation sensor but is difficult to observe in practice. Sudden step changes can occur if an IMU is subject to large variations or shocks. In our application, the platform is subject to slow changes in orientation and the IMU acquires small angle variations. The non-precise knowledge of the position of the beacons is also due to an incorrect IMU measurement.

### 2.3. Ranging Error Sources

**Figure 5:**
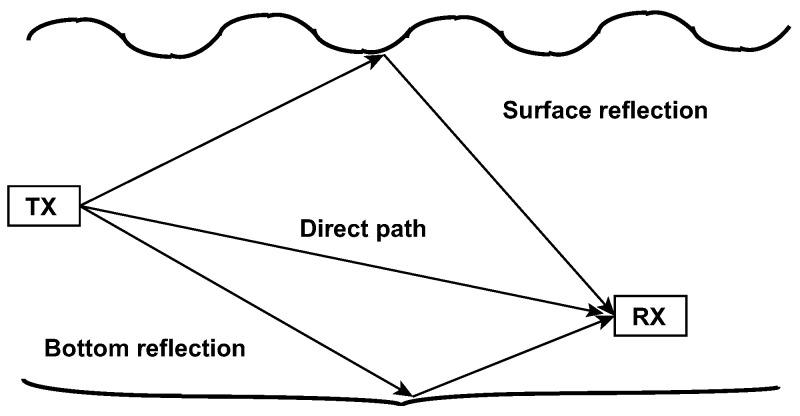
Ranging error sources: Multipath propagation.

**Figure 6:**
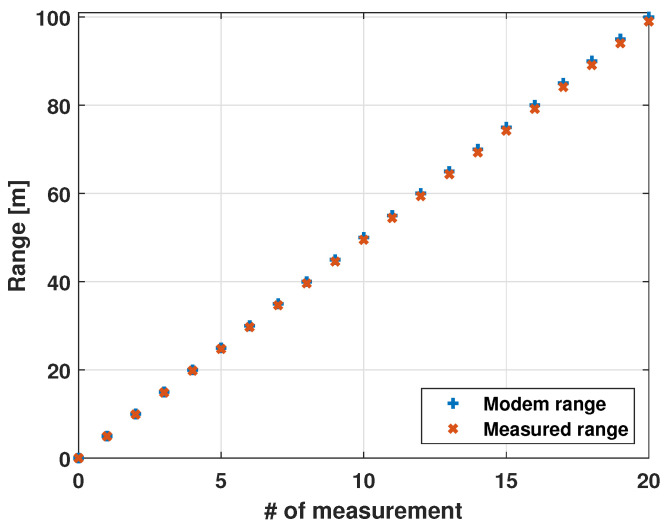
Range estimation [10].

The range computed by the One-Way Time Travel technique, with the clocks synchronized, is not affected by the errors of the GPS positioning (due to GPS time synchronization) or the IMU and it depends only on the sound propagation model used and its approximation (Figure 5). The Seamodem allows one to estimate the sound speed through a built-in procedure by knowing the exact distance between two modems. The sound speed is measured deploying two modems at a fixed distance (i.e., 10 m). Various tests were carried out. The tests involved two modems: The first one was kept fixed and the other one was moved 5 m apart at a time, along a straight path of 100 m. The range measured with the SeaModem was compared with the measurements of a laser meter (Figure 6). The sources of error in ranging can be: The varying nature of the sound of speed in the water column, the non-perfect synchronization of the clocks, the drift of each clock, and the delay in the computation by the microprocessor. In fact, all errors in acoustic system due to multipath, waves, biological, and anthropogenic activity generate a packet loss or an error in the range calculation.

## 3. Extended Kalman Filter

The data about the target (such as the ranges and the depth from the depth sensor) are merged together by an EKF that runs in a low power commercial embedded computer. The EKF is a nonlinear version of the Kalman filter which linearizes about an estimate of the current state. It is widely used in applications as inertial navigation systems. The filter works as a data fusion algorithm to compute the position of the target. The main elements of the EKF are:

- The estimated state vector: Usually includes only the variables of the system we want to know;
- The covariance matrix: Is a measure of estimation uncertainty. The matrix takes into account how sensor noise and dynamic uncertainty contribute to uncertainty about the estimated system state;
- The Kalman gain matrix: Used for correcting the estimate of the state of the system.

The essential Kalman filter equations are summarized as follows: [22]:

(1) $$\begin{array}{cccc} \; & \; & & \mathrm{Predictor}\;(\mathrm{Time}\mathrm{Updates})\\ {\hat{x}}_{k}\left(-\right) & = & \Phi_{k}{\hat{x}}_{k-1}\left(+\right) & \leftarrow \mathrm{Predicted}\mathrm{state}\mathrm{vector}\\ P_{k}\left(-\right) & = & \Phi_{k}P_{k-1}\left(+\right)\Phi_{k}^{T}+Q_{k-1} & \leftarrow \mathrm{Predicted}\mathrm{covariance}\mathrm{matrix}\\ \; & \; & & \mathrm{Corrector}\;(\mathrm{Measurements}\mathrm{Updates})\\ {\bar{K}}_{k} & = & P_{k}\left(-\right)H_{k}^{T}{\left(H_{k}P_{k}\left(-\right)H_{k}^{T}+R_{k}\right)}^{-1} & \leftarrow \mathrm{Kalman}\mathrm{gain}\\ {\hat{x}}_{k}\left(+\right) & = & {\hat{x}}_{k}\left(-\right)+{\bar{K}}_{k}\left(z_{k}-H_{k}{\hat{x}}_{k}\left(-\right)\right) & \mathrm{Corrected}\mathrm{state}\mathrm{estimate}\\ P_{k}\left(+\right) & = & P_{k}\left(-\right)-{\bar{K}}_{k}H_{k}P_{k}\left(-\right) & \mathrm{Corrected}\mathrm{covariance}\mathrm{matrix} \end{array}$$

These terms assume the following common meaning:

- $H_{k}$ is the measurement sensitivity matrix;
- $H_{k}{\hat{x}}_{k}$ is the predicted measurement;
- $z_{k}-H_{k}{\hat{x}}_{k}\left(-\right)$ is the innovations vector;
- ${\bar{K}}_{k}$ is the Kalman matrix gain;
- $P_{k}\left(-\right)$ is the predicted value of estimation covariance;
- $P_{K}\left(+\right)$, is the corrected value of estimation covariance;
- $R_{K}$, is the covariance matrix of sensor noise;
- ${\hat{x}}_{k}\left(-\right)$ is the predicted value of the estimated state vector.
- ${\hat{x}}_{k}\left(+\right)$ is the corrected value of the estimated state vector.
- $z_{K}$ is the measurement vector;
- $\Phi_{k}$ is the state transition matrix.

In nonlinear systems, the EKF characteristics equations are given by:

(2) $$\left\{\begin{array}{ccc} {\hat{x}}_{k}\left(+\right) & = & f({\hat{x}}_{k}\left(-\right))\\ H_{k} & = & {\left.\frac{\partial h(x)}{\partial x}\right|}_{x={\hat{x}}_{k}\left(+\right)} \end{array}\right.$$

The (6×1) estimated state vector $x_{k}$, which contains the variables we want to estimate is:

(3) $$x_{k}=\left[\begin{array}{c} pos_{X}\\ vel_{X}\\ pos_{Y}\\ vel_{Y}\\ pos_{Z}\\ vel_{Z} \end{array}\right]$$

where $pos_{i}$ and $vel_{i}$ indicate, respectively the coordinate *i* of the target and the velocity in direction *i* of the target, for i=X,Y,Z. The input of the EKF are the range from each SBL beacon to the receiver transducer $\left(z_{1},z_{2},z_{3},z_{4}\right)$ and the depth from the depth sensor on the receiver tablet $\left(z_{5}\right)$. The EKF matrices are as follows:

(4) $$\Phi =\left[\begin{array}{cccccc} 1 & \Delta t & 0 & 0 & 0 & 0\\ 0 & 1 & 0 & 0 & 0 & 0\\ 0 & 0 & 1 & \Delta t & 0 & 0\\ 0 & 0 & 0 & 0 & 1 & \Delta t\\ 0 & 0 & 0 & 0 & 0 & 1 \end{array}\right]$$

(5) $$H_{k}=\left[\begin{array}{cccccc} \frac{pos_{x,K}-s_{1,X}}{range(x,s_{1})} & 0 & \frac{pos_{y,K}-s_{1,Y}}{range(x,s_{1})} & 0 & \frac{pos_{z,K}-s_{1,Z}}{range(x,s_{1})} & 0\\ \frac{pos_{x,K}-s_{2,X}}{range(x,s_{2})} & 0 & \frac{pos_{x,K}-s_{2,X}}{range(x,s_{2})} & 0 & \frac{pos_{x,K}-s_{2,X}}{range(x,s_{2})} & 0\\ \frac{pos_{x,K}-s_{3,X}}{range(x,s_{3})} & 0 & \frac{pos_{x,K}-s_{3,X}}{range(x,s_{3})} & 0 & \frac{pos_{x,K}-s_{3,X}}{range(x,s_{3})} & 0\\ \frac{pos_{x,K}-s_{4,X}}{range(x,s_{4})} & 0 & \frac{pos_{x,K}-s_{4,X}}{range(x,s_{4})} & 0 & \frac{pos_{x,K}-s_{4,X}}{range(x,s_{4})} & 0\\ 0 & 0 & 0 & 0 & 1 & 0 \end{array}\right]$$

(6) $$z_{K}=\left[\begin{array}{c} range_{1,k}\\ range_{2,k}\\ range_{3,k}\\ range_{4,k}\\ depth_{k} \end{array}\right]=\left[\begin{array}{c} range_{1}\left(K-K\%n+1\right)\\ range_{2}\left(K-\left(K+3\right)\%n+1\right)\\ range_{3}\left(K-\left(K+2\right)\%n+1\right)\\ range_{4}\left(K-\left(K+1\right)\%n+1\right)\\ depth_{k} \end{array}\right]$$

(7) $$R=\left[\begin{array}{ccccc} s_{dr_{1}} & 0 & 0 & 0 & 0\\ 0 & s_{dr_{2}} & 0 & 0 & 0\\ 0 & 0 & s_{dr_{3}} & 0 & 0\\ 0 & 0 & 0 & s_{dr_{4}} & 0\\ 0 & 0 & 0 & 0 & s_{drz} \end{array}\right]$$

The relations between the ranges and the state of the system are not linear. In the $z_{k}$ vector, *K* represents time instant and *n* represents the total number of beacons in the system. As an example, for *n* to equal to 4, if the range 4 was acquired at time instant 1, it will be now updated at time instant 5. The elements on the diagonal of the R matrix are the standard deviations of the instrumentation. The *Q* and *P* matrices are tuned by trials. In our application the model of the diver is assumed to be a constant acceleration motion because of the non-precise knowledge of their motion.

It is also assumed that the categories within the time variation of all errors modeled in the Kalman filter will have to be classified as [6]:

- Systematic errors;
- White Gaussian Noise;
- Markov processes.

For example, a constant acceleration error leads to a velocity error that grows with time. Real navigation system errors do not fall exactly into these categories but can be approximated to them.

## 4. Simulation Environment

This section is devoted to illustrate the main features of the designed simulation environment. The simulation software has been designed in GNU Octave (https://www.gnu.org/software/octave/index) using the proprietary localization algorithms developed by Applicon S.r.l.. In details, the simulator allows one to recreate a fully understood application scenario knowing its main characteristics:

- Baseline between the transponders;
- Errors of the instrumentation;
- Approximation of the physical models of the sound propagation;
- Running and elaboration time of the process unit;
- Estimation algorithm.

**Figure 7:**
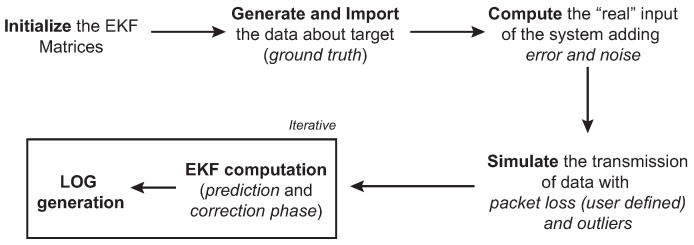
Simulation process scheme of the underwater localization system.

Thanks to the designed simulator, it is possible to run multiple experiments according to the scheme depicted in Figure 7, being efficient in terms of time and cost, compared to real experiments.

**Figure 8:**
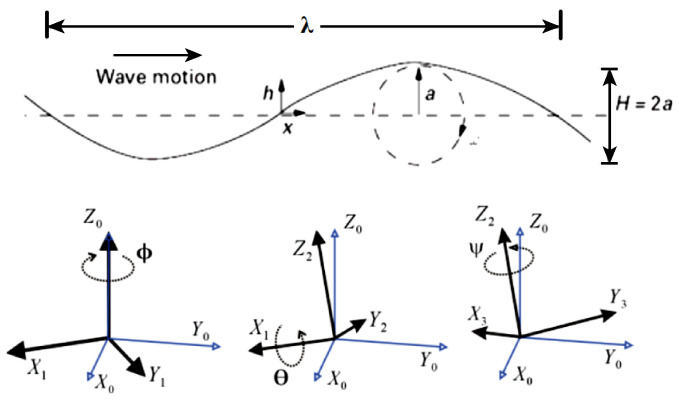
Wave motion.

The simulator implements the wave motion (see Figure 8) that affects the pose of the attached floating platform, the positioning installation errors, the instrumentation errors, the occurrence of outliers, and the packet losses. The main features of interest for the analysis are:

- The length of the baseline;
- The sampling time between measurements;
- The instrumentation errors (GPS, IMU, depth sensor, ranging);
- The ratio between the time at which the EKF estimation occurs and the time at which a measurement is acquired (speed of the EKF filter as multiple of time step of acquired measurement).

These parameters represent the aspects of the system that the designer can modify quickly to test new solutions.

The simulator consists in an algorithm that generates the input data for the EKF at each time step. In order to reproduce a realistic scenario, it is necessary to model the environment effects on the system. As an example, the wave motion was implemented because it was found to be useful to simulate the corresponding undesired change of position and orientation of the platform. In this respect, the roll, pitch, and yaw angles of the floating platform are modeled as:

(8) $$\begin{array}{ccc} w_{roll}\left(t\right) & = & A_{roll}*sin\left(2\pi f_{roll}t+\phi_{roll}\right)\\ w_{pitch}\left(t\right) & = & A_{pitch}*sin\left(2\pi f_{pitch}t+\phi_{pitch}\right)\\ w_{yaw}\left(t\right) & = & A_{yaw}*sin\left(2\pi f_{yaw}t+\phi_{yaw}\right). \end{array}$$

**Figure 9:**
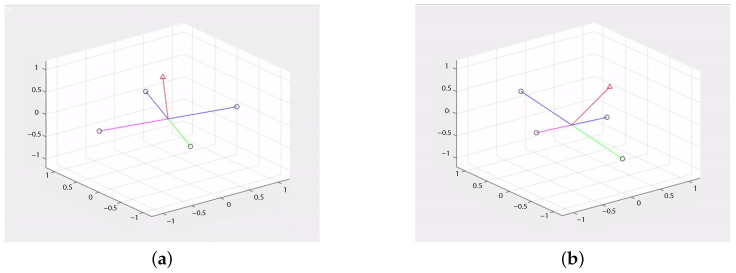
Simulated platform motion due to wave effect: wave height equals to 0.2[m] (**a**) and wave height equals to 1.0[m] (**b**).

Different levels of wave magnitude were tested based on the wave height of the Beaufort scale [23] and the values of roll, pitch, and yaw are inside a range whose minimum and maximum values are selected according to the mentioned Beaufort scale. Figure 9 shows platform inclination for different values of wave height: Figure 9a refers to a wave height equals to 0.2[m] (level 2 of Beaufort scale) whilst Figure 9b refers to a wave height equals to 1.0[m] (level 4 of Beaufort scale).

Moreover, modeling the occurrence of packet loss and the presence of outliers reproduces some of the troubles that could derive from the underwater environment. In particular, the packet loss and outliers are modeled using two random noise Gaussian distribution with zero mean 0 and unitary variance. As an example, for the case of the packet loss, if the value of random function is less than a threshold, the information is considered missed. In shallow water or when there are buildings/rock or other obstacles, it is possible to have multipath propagation instead of a direct propagation. Without a direct propagation of the signal, the information transmitted will be wrong and the localization estimates will feature some level of degradation too. The ranges computed, due to signal reflection, could be larger than the true range computed when the propagation is direct. These aspects have been included in the simulator by accounting random Gaussian distribution with zero mean 0 and variance 1, to dispose of more realistic scenarios.

### Simulation Environment Validation

**Figure 10:**
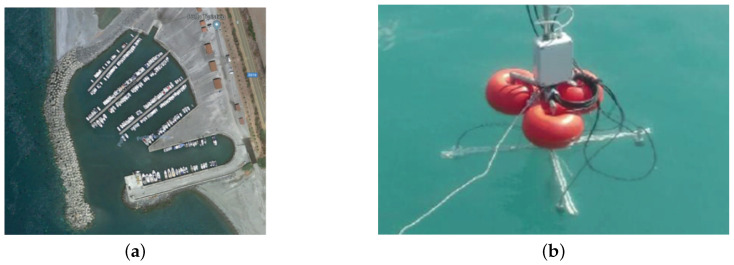
Touristic harbor of Campora San Giovanni, Cosenza, Italy (**a**) and SBL platform with four beacons (**b**).

In order to demonstrate the effectiveness of the designed EKF an extensive campaign of tests have been performed by accounting the simulation environment described in Section 4. The output of the simulator is matched with the data collected in a real underwater diving. The accounted scenario refers to the touristic harbor of Campora San Giovanni, Cosenza, Italy (see Figure 10a) and is characterized by the following conditions:

- Sea conditions of level 2 on the Beaufort scale [23];
- Depth of the seafloor of about 3.5 m with 50% of packet loss;
- Sound propagation speed of 1500 m per seconds.

As described in Section 2, we consider a floating platform (the SBL base) with four transducers fixed on a cross (see Figure 10b). The floating platform is moored on the seafloor. The target is the receiver embedded with an underwater tablet and used by the diver to localize themselves in the area of interest. In practice, in order to carry out more precise tests, a surface vehicle was appropriately instrumented to simulate the movement of the diver. Specifically, a differential GPS was mounted on the vehicle, while the SBL receiver was mounted two meters deep under the same vehicle, just below the GPS, using a rigid rod. In this way the two systems have the same local XY reference system and can be easily compared. The use of a fixed Z coordinate does not affect the goodness of the tests as a pressure sensor is present on the SBL receiver and therefore the Z coordinate was already measurable, unlike the X and Y coordinates.

**Remark** **1.** *It is important to note that the accounted scenario can be compared with typical archaeological sites in shallow water. In fact, many Italian archaeological sites are in shallow water with sandy seabed as the underwater archaeological park of Baiae (Naples—Italy). Furthermore, the packet loss percentage is of the same entity of the simulated percentage because of the presence of obstacles that can be considered as archaeological assets.*

The simulation environment has been validated by taking into account real data acquired during a diving session. In particular, three validation sessions have been undertaken by considering the simulator to have the same physical parameters as the hardware system: The first one simulates a session where a diver moves on a square trajectory, while the second session consists of a diver moving on a spiral trajectory and the latter session of a diver moving along a random trajectory. The considered reference trajectory is compared with the data acquired by the instrumentation and with the EKF estimates. The following positioning error has been considered:

(9) $$e_{pos}\left(t\right)=\sqrt{{\left(x_{true}\left(t\right)-x_{j}\left(t\right)\right)}^{2}+{\left(y_{true}\left(t\right)-y_{j}\left(t\right)\right)}^{2}+{\left(z_{true}\left(t\right)-z_{j}\left(t\right)\right)}^{2}},\;\;j=1,2$$

where the index *j* alternatively indicates the instrumentation measures (j=1) and the EKF estimates (j=2). Finally in order to perform a numerical analysis the mean and standard deviation values of the position error ($e_{tot}$) have been computed and reported in Table 2.

**Figure 11:**
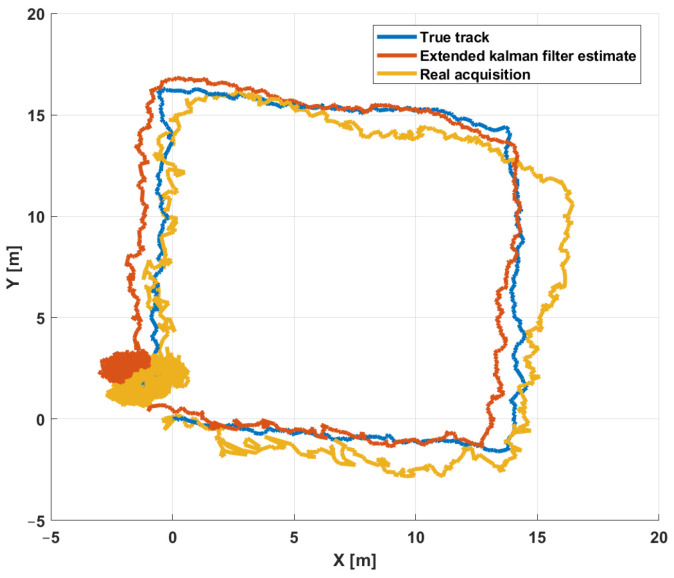
Simulation environment validation: Comparison between trajectory (blue line), EKF estimate (red line), and instrumentation acquisition (yellow line).

Figure 11 shows one of the different tests carried out in the harbor. The figure compares the real path (blue line), the path estimated with the hardware instrumentation (yellow line), and the one estimated by the simulator (red line). The real path was measured using a differential GPS mounted on a surface vehicle that simulates the diver’s movement. The real acquisition path is estimated using the SBL receiver installed under the vehicle at a depth of two meters. Finally, the EKF estimate path is computed by the simulator considering the same physical parameters of the hardware system.

## 5. Simulation Results and Statistical Analysis

The idea behind the use of proposed simulator is to reduce the mean and standard deviation values of the SBL system (SBL base + SBL receiver) positioning error (Equation (9)) reported in Table 2 (case j=1). Three simulation scenarios have been considered with the target (the diver) moving along a defined path (circular, spiral, and random path) and with the platform (the SBL base) moored in a geo-referenced point.

The simulation tests:

- Two levels for the standard deviation of the measurement of GPS, IMU, Range, and Depth Sensor;
- Two levels for the length of the baseline;
- Two levels for the sampling time of the measurements;
- Two levels for the packet loss probability.

The levels are summarized in the following table:

### 5.1. Scenario 1

**Figure 12:**
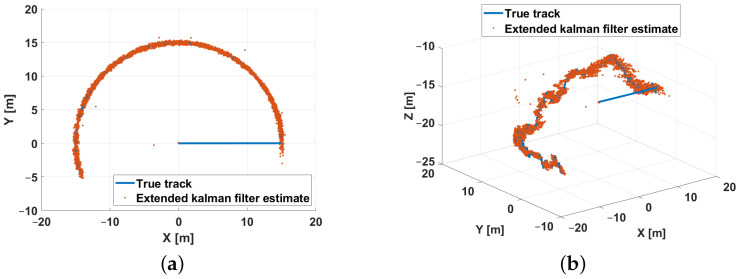
Scenario 1—Reference trajectory: *XY* (**a**) and *XYZ* (**b**) views.

In the first scenario a semicircle with radius 15 [m] has been considered as a reference trajectory. Figure 12 respectively shows the *XY* (Figure 12a) and the *XYZ* (Figure 12b) trajectory views. The trajectory is defined in the discrete domain, each point is sampled with a *T* sampling period. At every time instant, the simulator adds noise to measurements in order to model the real instrumentation of the system. The input for the EKF are the measurements of the ranges and the depth according to a round-robin scheduling logic. For each combination of the parameters reported in Table 3 ($2^{6}$ possible combinations), 50 simulations were run for a total of 6400 simulations. Furthermore, in order to evaluate the performance of the designed EKF, the following errors, characterizing respectively the estimation errors along the X, Y, and Z axis and the total position error, have been used as performance indexes.

(10) $$\begin{array}{c} e_{x}\left(t\right)=x_{true}\left(t\right)-x_{EKF}\left(t\right)\\ e_{y}\left(t\right)=y_{true}\left(t\right)-y_{EKF}\left(t\right)\\ e_{z}\left(t\right)=z_{true}\left(t\right)-z_{EKF}\left(t\right)\\ e_{tot}\left(t\right)=\sqrt{e_{x}{\left(t\right)}^{2}+e_{y}{\left(t\right)}^{2}+e_{z}{\left(t\right)}^{2}} \end{array}$$

**Figure 13:**
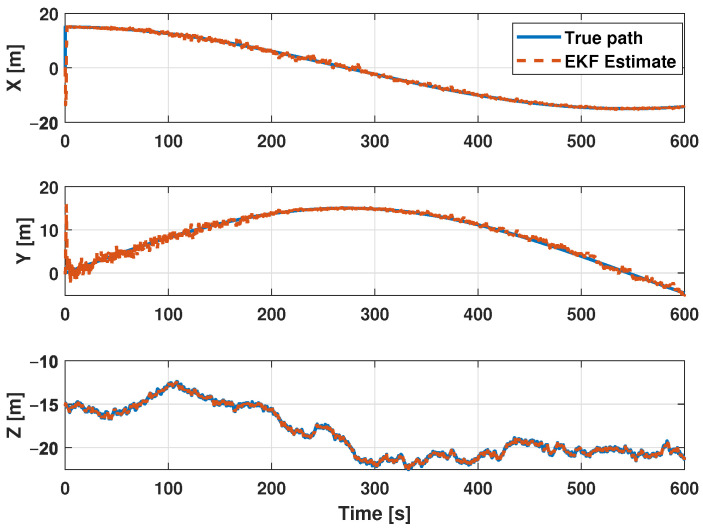
Scenario 1—Comparison between true path (blue line) and EKF estimate (dot red) along X, Y, and Z axes.

**Figure 14:**
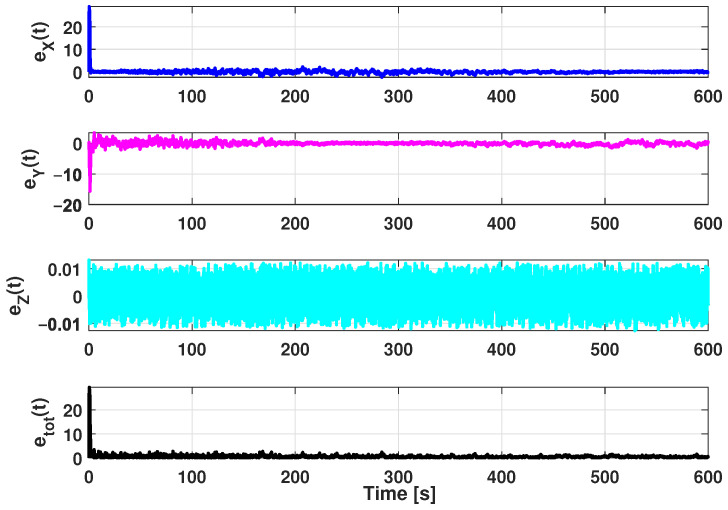
Scenario 1—Estimation error along X, Y, and Z axes and total position error.

The estimation procedure results related to this first scenario are reported in Figure 13 and Figure 14. In particular, in Figure 13, a comparison between the reference trajectory and the EKF estimate along the X, Y, and Z axes is reported. On the other side, Figure 14 depicts the estimation errors and the total position error $e_{x}\left(t\right)$, $e_{y}\left(t\right)$, $e_{z}\left(t\right)$, and $e_{tot}\left(t\right)$. Furthermore, for a more quantitative comparison it is possible to refer to Table 4 where the errors Equation (10) averaged along the simulation period are reported.

**Figure 15:**
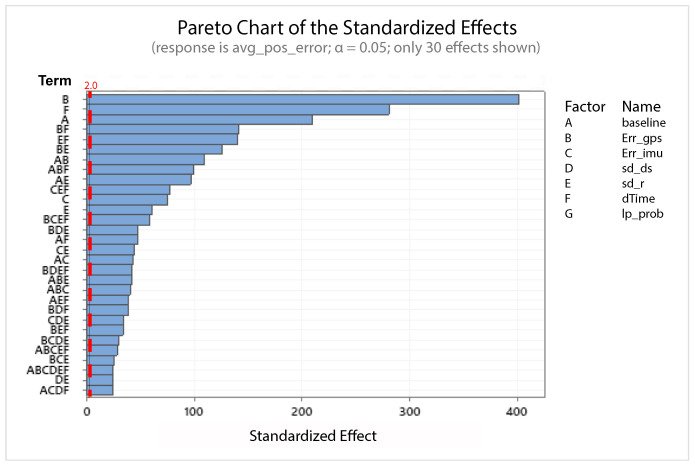
Scenario 1—Pareto plot for the RMS (Root-Mean-Square) positioning error.

**Figure 16:**
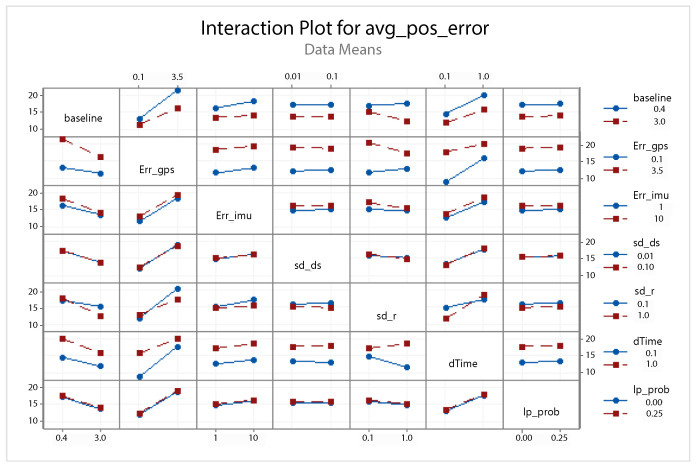
Scenario 1—Interaction plot for the RMS positioning error.

**Figure 17:**
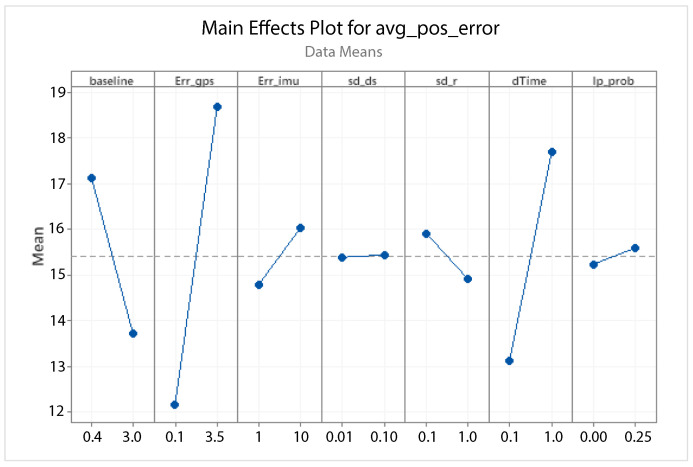
Scenario 1—Main effect plot for the RMS positioning error.

**Figure 18:**
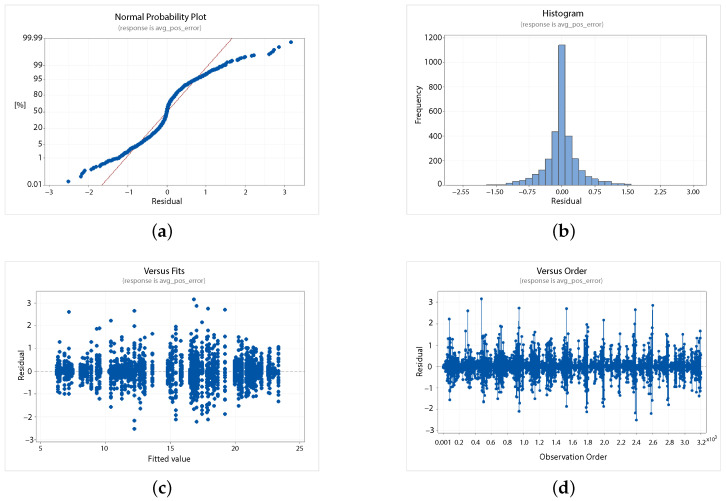
Scenario 1—Residuals plot for the RMS positioning error: normal probability (**a**), histogram (**b**), residuals versus fits (**c**) and residuals versus order (**d**) plots.

A statistical 2-level factorial analysis [24,25] has been conducted on the simulation outputs in order to evaluate how the variations in parameter (factor) levels influence the total positioning error $e_{tot}\left(t\right)$. In statistics, a full factorial experiment is an experiment whose design consists of two or more factors, each one with discrete possible values or “levels”, and whose experimental units take on all possible combinations of these levels across all such factors. The response is the Root-Mean-Square (RMS) positioning error and the main aspects of interest are the interaction between the parameters and the main effects of each factor for the final positioning error. The results of the statistical 2-level factorial analysis, referred to the factor levels reported in Table 3, are shown in Figure 15, Figure 16, Figure 17 and Figure 18, where are respectively reported: The Pareto plot of the RMS positioning error, the interaction plot of the RMS positioning error, the main effects plot for the RMS positioning error, and the residual plots for the RMS positioning error.

In Figure 15 the Pareto plot is reported. It represents a chart that shows the absolute values of the standardized effects (the standardized effects are t-statistics that test the null hypothesis that the effect is 0 [26,27]) from the largest effect to the smallest one. This kind of plot allows one to determine the magnitude and the importance of the effects. The chart also plots a reference line (the dotted red line in the Figure 15) to show which effects are statistically significant. In details, the bars that cross the reference line are statistically significant. Figure 16 depicts the interaction plot that shows how the relationship between one categorical factor and a continuous response depends on the value of the second categorical factor. This plot displays the means for the levels of one factor on the x-axis and a separate line for each level of another factor. If the plot shows parallel lines then no interactions occur, otherwise an interaction occurs and the more the lines are non-parallel, the greater the strength of the interaction is. The main effects plot are, instead, displayed in Figure 17. This plot shows the means for each group within a categorical variable. The main effects plot is obtained by plotting the means for each value of a categorical variable and by connecting them trough a line. A dotted line is also drawn as a reference line at the overall mean. One can look at the line to determine whether a main effect is present for a categorical variable. If the line is parallel to the x-axis, there is no main effect present and the response mean is the same across all factor levels. On the contrary, if the line is not horizontal there is a main effect present and the response mean is not the same across all factor levels. The steeper the slope of the line, the greater the magnitude of the main effect. Figure 18 shows the residual plots the are graphs that are used to examine the goodness-of-fit in regression. Examining residual plots helps one to determine whether the ordinary least squares assumptions are being met. If these assumptions are satisfied, then ordinary least squares regression will produce unbiased coefficient estimates with the minimum variance. In particular, Figure 18a shows normal probability plot of residuals to verify the assumption that the residuals are normally distributed, Figure 18b depicts the histogram of residuals to determine whether the data are skewed or whether outliers exist in the data, Figure 18c shows residuals versus fits to verify the assumption that the residuals have a constant variance and Figure 18d depicts the residuals versus order plot to verify the assumption that the residuals are uncorrelated with each other. The performed statistical 2-level factorial analysis highlights that the most relevant results are that reported in the Pareto chart and in the main effects plots. In fact, from these figures it is evident that the estimation accuracy is highly influenced by factors of GPS accuracy, baseline length, and sampling time.

### 5.2. Scenario 2

**Figure 19:**
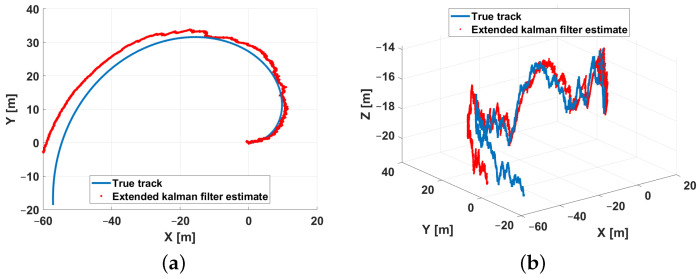
Scenario 2—Reference trajectory: *XY* (**a**) and *XYZ* (**b**) views.

**Figure 20:**
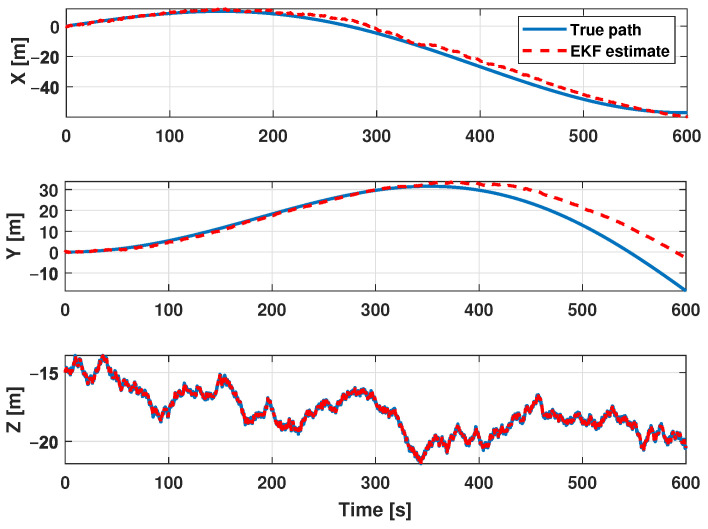
Scenario 2—Comparison between true path (blue line) and EKF estimate (dot red) along X, Y, and Z axis.

**Figure 21:**
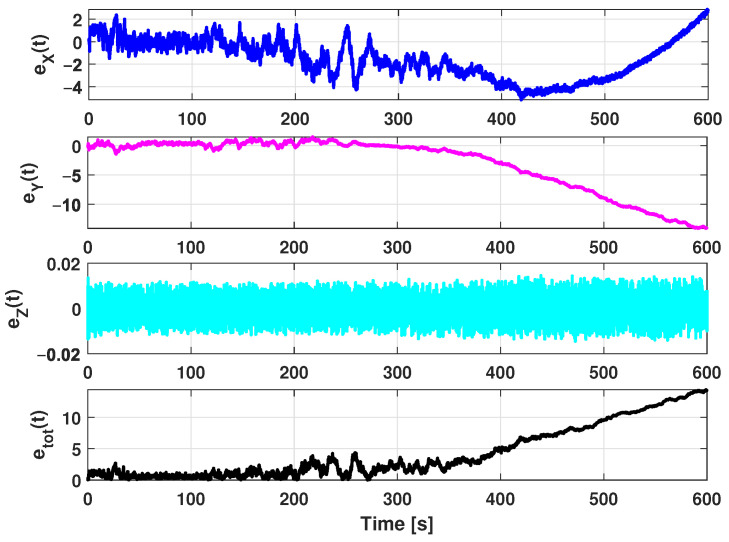
Scenario 2—Estimation error along X, Y, and Z axes and total position error.

In the second scenario a spiral has been used as a reference trajectory. Figure 19 respectively shows the *XY* (Figure 19a) and the *XYZ* (Figure 19b) trajectory views. It is important to highlight that the spiral trajectory allows one to highlight the influence of the (increasing) distance of the target from the center of the beacons’ cross. The trajectory is defined in the discrete domain where each point is sampled with a *T* sampling period. At every time instant, the simulator adds noise to measurements to reproduce the real instrumentation of the system. The input for the EKF are the measurements of the ranges and depth according to a round-robin scheduling logic. In addition, in this scenario, for each combination of parameters reported in Table 3 ($2^{6}$ possible combinations), 50 simulations were run for a total of 6400 simulations. Furthermore, in order to evaluate the performance of the designed EKF, the estimation and position errors (Equation (10)) have been accounted as performance indexes. The estimation procedure results related to this scenario are reported in Figure 20 and Figure 21. Figure 20 shows a comparison between the reference trajectory and the EKF estimate along the X, Y, and Z axes, whilst Figure 21 depicts the estimation errors and the total position error $e_{x}\left(t\right)$, $e_{y}\left(t\right)$, $e_{z}\left(t\right)$, and $e_{tot}\left(t\right)$. Furthermore, in Table 5 the average value of estimation and position errors (Equation (10)) are reported. From these results it is evident that in this scenario the designed EKF also exhibits good performance.

**Figure 22:**
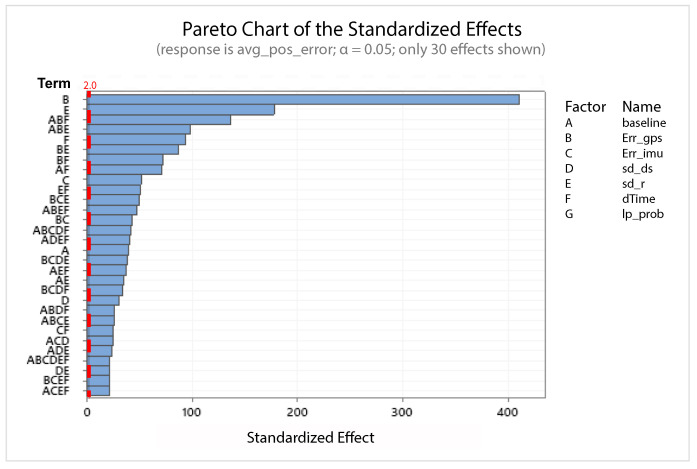
Scenario 2—Pareto plot for the RMS positioning error.

**Figure 23:**
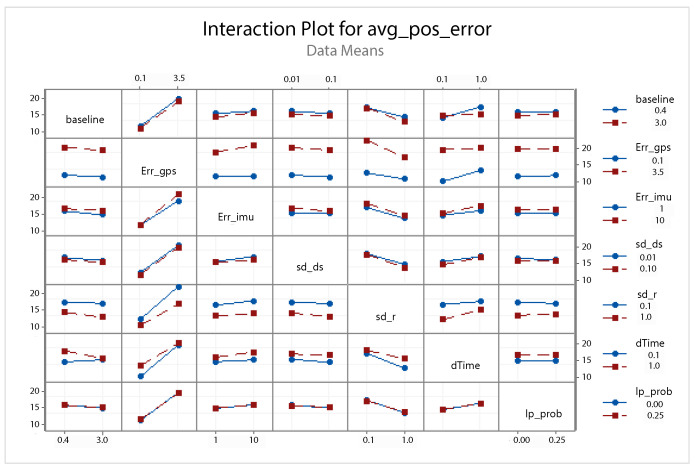
Scenario 2—Interaction plot for the RMS positioning error.

**Figure 24:**
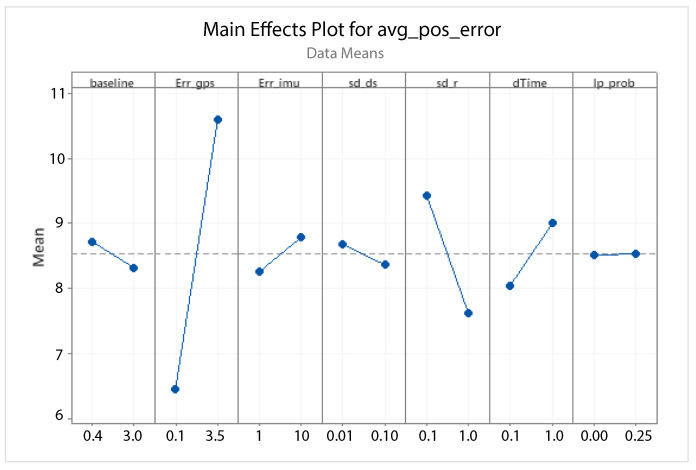
Scenario 2—Main Effect Plot for the RMS positioning error.

Additionally, for this scenario a statistical 2-level factorial analysis have been conducted and the related results (see Figure 22, Figure 23 and Figure 24) are comparable with those obtained in the previous scenario.

### 5.3. Scenario 3

**Figure 25:**
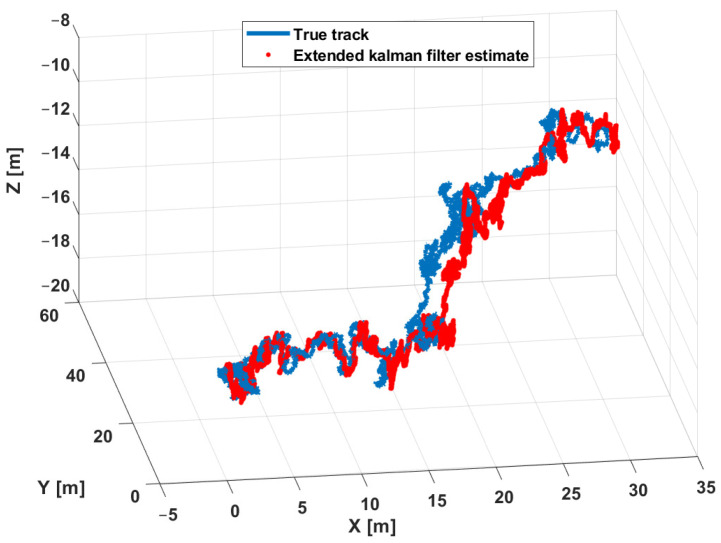
Scenario 3—XYZ view.

In this latter scenario, a random trajectory has been considered as a reference path. This kind of trajectory is useful to stress the system because it allows one to test conditions where the target suddenly changes the moving direction. Figure 25 reports the *XYZ* trajectory views. The trajectory is defined in the discrete domain and each point is sampled with a *T* sampling period. At every time instant, the simulator adds noise to measurements to reproduce the real instrumentation of the system. The input for the EKF are the measurements of the ranges and depth according to a round-robin scheduling logic. In addition, for this case, for each combination of parameters reported in Table 3 ($2^{6}$ possible combinations), 50 simulations were run for a total of 6400 simulations.

**Figure 26:**
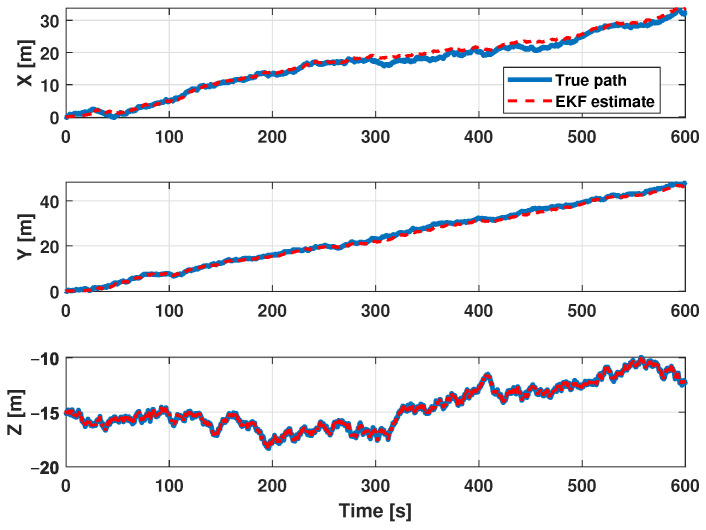
Scenario 3—Comparison between true path (blue line) and EKF estimate (dot red) along X, Y, and Z axes.

**Figure 27:**
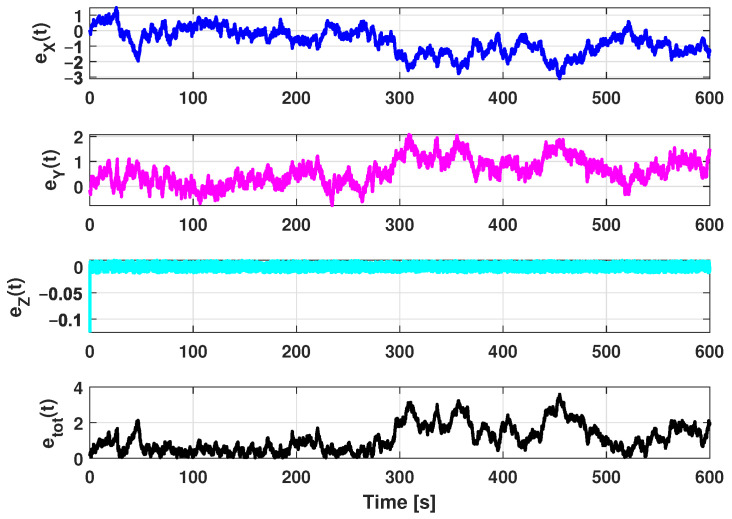
Scenario 3—Estimation error along X, Y, and Z axes and total position error.

In Figure 26 a comparison between the reference trajectory and the EKF estimate along the X, Y, and Z axes is reported. Figure 27 shows the estimation errors and the total position error $e_{x}\left(t\right)$, $e_{y}\left(t\right)$, $e_{z}\left(t\right)$, and $e_{tot}\left(t\right)$. In Table 6 the average value of estimation and position errors (Equation (10)) are reported.

**Figure 28:**
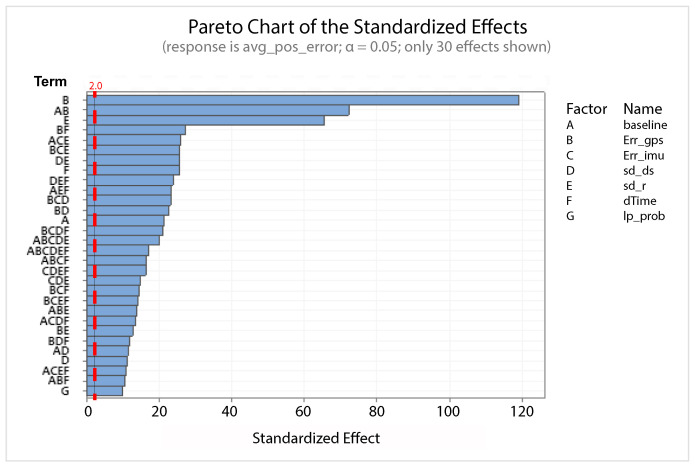
Scenario 3—Pareto plot for the RMS positioning error.

**Figure 29:**
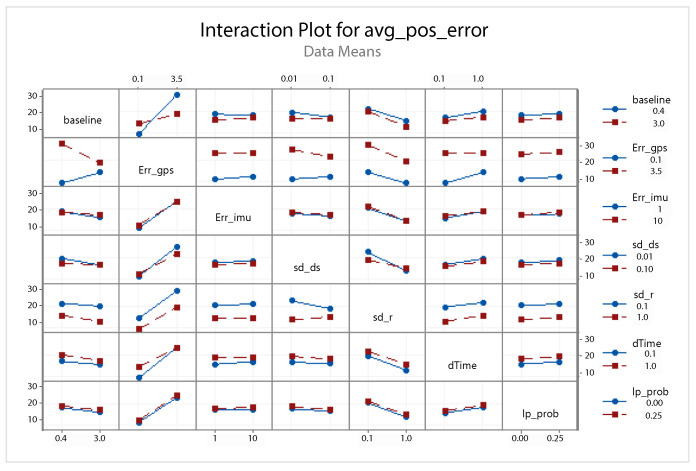
Scenario 3—Interaction plot for the RMS positioning error.

**Figure 30:**
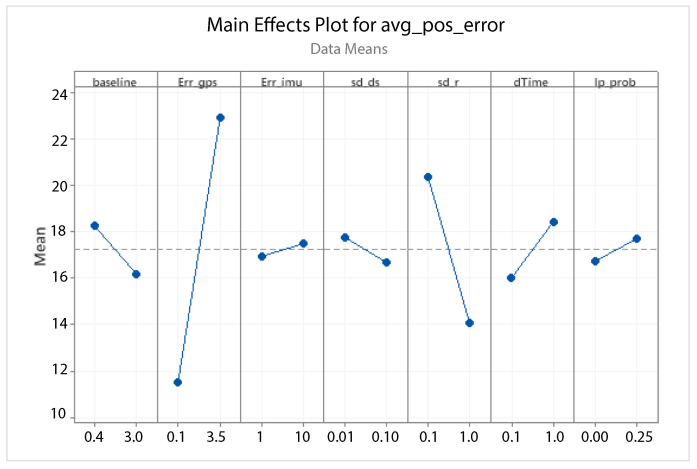
Scenario 3—Main effect plot for the RMS positioning error.

From these results it is evident that the designed EKF exhibits good performance. In fact, it can be observed that in this scenario, a good performance was also achieved in terms of bounded estimation error. Furthermore, from the results of statistical 2-level factorial analysis reported in Figure 28, Figure 29 and Figure 30 it is possible to state that:

- The length of the baseline is significant to increase the accuracy of the positioning system;
- A good GPS receiver permits to achieve better results. The GPS has relevant interaction with the other parameters;
- A large sampling period means no congruent range measurements. If a measured range is connected to a relative position of the platform in respect to the target at a time instant kT, the next measured range at time (k+1)T regards a too different relative position of the platform in respect to the previous one. This difference increases with the length of the sampling period *T*. In fact, the data fusion inside the EKF takes place by trying to estimate the position of the target at a certain time instant with data collected at different time instants.

## 6. Conclusions

This paper has presented a simulation and statistical analysis of the sources of error for an acoustic underwater localization system. The main purpose was to identify the parameters of the localization system that mainly affect to total localization error. Such an analysis is instrumental in the effort to optimize the accuracy of the position estimates. The studies proved that a significant influence was due to the length of the baseline. The increase of the baseline, reaching up to the typical lengths of a LBL configuration, permitted an improvement in accuracy but also introduced issues related to the difficulty of deployment and various complications (mechanical, manufacturing, and portability). The error in the ranging should not be neglected, especially with the increase of the distance from the center of the cross, when the segments from the beacons to the target become almost parallel to each other. This source of error was accentuated when the sampling period increased because of the distortion of the area in which the target could be individuated. The sampling period between two successive acquisitions should be as small as possible to reduce the inconsistency in the range measurements. In fact, an old measurement is not as effective as a new one and the information carried in the filter is not effective enough for the estimation of the actual position of the target.

The results obtained with the simulator allowed us to improve the performance of the actual SBL system. In fact, before applying the optimization procedure, the total positioning error, as reported in Table 2, was about two meters on average over the various tests performed. The optimizations undertaken by the simulators made it possible to reduce the total error by about one meter, as reported in Table 4, Table 5 and Table 6.

## Figures and Tables

**Table 1:** Inertial Measurement Unit (IMU) bias.

IMU Grade	Accelerometer Bias		Gyro Bias	
	mg	m s${}^{-2}$	${}^{\circ }$hr${}^{-1}$	rad s${}^{-1}$
Marine	0.01	$10^{-4}$	0.001	$5\times 10^{-9}$
Aviation	0.03–0.1	$3\times 10^{-4}–10^{-3}$	0.01	$5\times 10^{-8}$
Intermediate	0.1–1	$10^{-3}–10^{-2}$	0.1	$5\times 10^{-7}$
Tactical	1–10	0.01–0.1	1–100	$5\times 10^{-6}–5\times 10^{-4}$
Consumer	>3	>0.03	>100	>$5\times 10^{-4}$

**Table 2:** Positioning error: Mean and standard deviation values for instrumentation error (j=1) and the Extended Kalman Filter (EKF) estimate (j=2).

Trajectory	j=1		j=2	
	Mean Value	Standard Deviation	Mean Value	Standard Deviation
Square	1.78	0.84	1.39	0.72
Spiral	1.72	0.81	1.38	0.71
Random	1.81	0.86	1.41	0.75

**Table 3:** Factor levels of the simulation parameters.

Parameter (Factor)	Type of Parameter	Levels	
		LOW	HIGH
Baseline	Design length of the support arm of each beacon [m]	±0.4	±3
GPS	Standard Deviation [m]	±0.1	±3.5
IMU	Standard Deviation ${[}^{\circ }]$	±1.0	±10
Depth Sensor	Standard Deviation [m]	±0.01	±0.1
Range	Standard Deviation [m]	±0.1	±1.0
Sampling Time	Time [s]	±0.1	±1.0
Packet Loss	Probability [%]	±0	±25

**Table 4:** Scenario 1—Tracking errors and total position error: Average value [m].

$e_{x}\left(t\right)$	$e_{y}\left(t\right)$	$e_{z}\left(t\right)$	$e_{\mathrm{tot}}\left(t\right)$
0.61	0.64	0.049	0.88

From these results it is evident that the designed EKF exhibits good performance.

**Table 5:** Scenario 2—Tracking errors and total position error: Average value [m].

$e_{x}\left(t\right)$	$e_{y}\left(t\right)$	$e_{z}\left(t\right)$	$e_{\mathrm{tot}}\left(t\right)$
0.61	0.63	0.049	0.88

**Table 6:** Scenario 3—Tracking errors and total position error: Average value [m].

$e_{x}\left(t\right)$	$e_{y}\left(t\right)$	$e_{z}\left(t\right)$	$e_{\mathrm{tot}}\left(t\right)$
0.92	0.95	0.049	1.32

## Data Availability

The data presented in this study are available on request from the corresponding author. The data are not publicly available due to confidential reason.
